# Hormone replacement therapy use and plasma levels of sex hormones in the Norwegian Women and Cancer Postgenome Cohort – a cross-sectional analysis

**DOI:** 10.1186/1472-6874-8-1

**Published:** 2008-01-14

**Authors:** Marit Waaseth, Kjersti Bakken, Vanessa Dumeaux, Karina S Olsen, Charlotta Rylander, Yngve Figenschau, Eiliv Lund

**Affiliations:** 1 Institute of Community Medicine, University of Tromsø, Norway; 2 Department of Genetics, Institute for Cancer Research, Rikshospitalet-Radiumhospitalet Medical Center, Montebello, Oslo, Norway; 3 Norwegian Institute for Air Research, Tromsø, Norway; 4 Department of Medical Biochemistry, University Hospital of North Norway, Tromsø, Norway

## Abstract

**Background:**

Hormone replacement therapy use (HRT) is associated with increased breast cancer risk. Our primary objective was to explore hormone levels in plasma according to HRT use, body mass index (BMI) and menopausal status. A secondary objective was to validate self-reported questionnaire information on menstruation and HRT use in the Norwegian Women and Cancer postgenome cohort (NOWAC).

**Methods:**

We conducted a cross-sectional study of sex hormone levels among 445 women aged 48–62 who answered an eight-page questionnaire in 2004 and agreed to donate a blood sample. The samples were drawn at the women's local general physician's offices in the spring of 2005 and sent by mail to NOWAC, Tromsø, together with a two-page questionnaire. Plasma levels of sex hormones and Sex Hormone Binding Globulin (SHBG) were measured by immunometry. 20 samples were excluded, leaving 425 hormone measurements.

**Results:**

20% of postmenopausal women were HRT users. The plasma levels of estradiol (E_2_) increased with an increased E_2 _dose, and use of systemic E_2_-containing HRT suppressed the level of Follicle Stimulating Hormone (FSH). SHBG levels increased mainly among users of oral E_2 _preparations. Vaginal E_2 _application did not influence hormone levels. There was no difference in BMI between HRT users and non-users. Increased BMI was associated with increased E_2 _and decreased FSH and SHBG levels among non-users. Menopausal status defined by the two-page questionnaire showed 92% sensitivity (95% CI 89–96%) and 73% specificity (95% CI 64–82%), while the eight-page questionnaire showed 88% sensitivity (95% CI 84–92%) and 87% specificity (95% CI 80–94%). Current HRT use showed 100% specificity and 88% of the HRT-users had plasma E_2 _levels above the 95% CI of non-users.

**Conclusion:**

Users of systemic E_2_-containing HRT preparations have plasma E_2 _and FSH levels comparable to premenopausal women. BMI has an influence on hormone levels among non-users. NOWAC questionnaires provide valid information on current HRT use and menopausal status among Norwegian women who are between 48 and 62 years old.

## Background

Plasma concentrations of steroid hormones influence the risk of breast cancer among both premenopausal and postmenopausal women, and estrogen is regarded as a carcinogen in cancer development [1,2]. Several epidemiological studies have examined female sex hormone levels, but hormone replacement therapy (HRT) users were either excluded [3-7] or they were not classified according to the type of HRT used [8,9]. One exception is a prospective case-control study nested within the Nurses' Health Study (NHS) [10]. However, it is uncertain whether results from the USA can be generalized to the Norwegian female population, due to different types of HRT preparation dominating the two markets, i.e. conjugated equine estrogens in the USA and micronized 17-β-estradiol or estradiol valerate in Norway. Several clinical studies have examined the relationship between HRT use and hormone levels, although in relatively small and highly selected populations [11,12]. Elevated estrogen levels may also be a result of high body mass index (BMI), through the conversion of androstenedione to estrone in adipose tissue [6,13].

The Norwegian Women and Cancer study (NOWAC) is a population-based, nation-wide cohort study which prospectively measures risk factors and biomarkers by means of repeat questionnaires and blood sample collection. The study has previously shown [14] that current use of HRT is associated with increased breast cancer risk, in agreement with similar observational studies like the Million Women Study (MWS)[15] and the European Prospective Investigation into Cancer and Nutrition (EPIC) [16]. It should be noted that breast cancer risk may differ according to differing HRT regimens [17], and that self-reported use of HRT and menstruation status among NOWAC participants has not been validated and described through hormone levels in plasma. The quality of results from research into questionnaire information depends heavily on the questions asked, and validation of the variables used is essential in this respect [18]. A cross-sectional descriptive study of hormone levels is also important, to avoid misclassification of subjects in a subsequent gene expression analysis of the same material.

In this study, we explore hormone levels in plasma according to HRT use, BMI and menopausal status. We further use plasma hormone levels to investigate the validity of self-reported information on menstruation, current HRT use and different HRT regimens in the NOWAC postgenome cohort.

## Methods

### Study population

The Norwegian Women and Cancer Study (NOWAC) is a cohort study based on questionnaires mailed to women who are 30–70 years old [19]. Participants are randomly drawn from the Central Population Register. From 1991 up until June 2007, 171 977 women had been enrolled in NOWAC, of whom 167 058 are still participating. Questionnaire information on diet, lifestyle and the use of medication is available, with 1–2 repeat measurements at 4–6 year intervals. The NOWAC postgenome cohort consists of 49 233 participants born between 1943 and 1957, who contributed a blood sample between 2003 and 2006. Written informed consent is obtained from each participant and the collection and storing of questionnaire information and blood samples is approved by The Regional Committee for Medical Research Ethics and the Norwegian Data Inspectorate. Statistics Norway obtains updated information on deaths and migration and performs the sampling of women, thereby providing a complete follow-up of participants.

**Figure 1:**
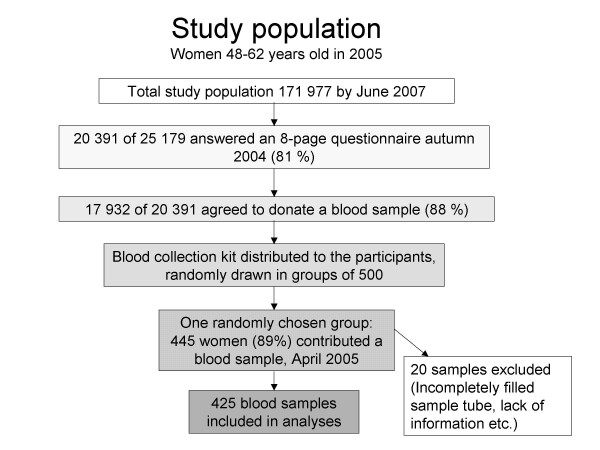
Flow chart of the study population.

The present study is a cross-sectional analysis within the NOWAC postgenome cohort (Figure 1). Of the 20 391 women who answered an eight-page questionnaire in the autumn of 2004 (response rate 81%), 17 932 agreed to donate a blood sample. Women, randomly drawn in groups of 500, were mailed a blood collection kit and an accompanying two-page questionnaire in April 2005. One reminder was mailed after three weeks to non-responders. The overall response rate was 74%. The two-page questionnaire included questions regarding menopausal status, smoking, weight, height, use of HRT, oral contraceptives or other medication, omega-3 intake, intake of soy or other dietary supplements, and details concerning blood specimen collection (date, hour, posture). Our present study included 445 responders from one group of 500 women (89%); 3.2% declined to participate, 0.8% had died or migrated and 7.0% did not respond. Six samples were excluded due to incompletely filled blood collection tubes. Additionally, 14 women were excluded due to a lack of information concerning menopausal status, use of HRT or type of HRT used. This left plasma sample measurements for 425 women.

### Collection and processing of blood samples

The blood samples were drawn at the women's local general physician's offices, using the blood collection kit. For collection of plasma and buffy coat we used a Vacuette^® ^Coagulation Tube (Greiner Bio-One GmbH, Kremsmünster, Austria) containing citrate buffer 0.109 mol/L (3.2%); 1 part citrate to 9 parts blood. For collection of RNA we used a PAXgene™ Blood RNA tube (PreAnalytiX GmbH, Hombrechticon, Switzerland), which is a BD Vacutainer™ containing a proprietary reagent that immediately stabilizes intracellular RNA. The samples were mailed overnight to the Institute of Community Medicine at the University of Tromsø, Norway. The women were requested not to have their blood samples drawn on Thursdays and Fridays, in order to avoid a weekend mail delay. The blood samples were generally received by the NOWAC biobank staff within 1–2 days (92%). Upon arrival, the Vacuette^® ^Coagulation Tubes were centrifuged at 3000 rpm for 15 minutes. Plasma (2 × 1.8 mL) and buffy coat (1.0 mL) were frozen at -20°C and subsequently transferred to -70°C within one week. PAXgene™ Blood RNA tubes were frozen directly at -20°C and transferred to -70°C without pre-processing. The results of gene expression analyses will be published at a later stage.

### Laboratory analysis

All the hormone analyses were performed at the Department of Medical Biochemistry, University Hospital of North Norway, Tromsø, Norway. Plasma levels of estradiol (E_2_), progesterone (P_4_) and Follicle Stimulating Hormone (FSH) were measured by immunometry, using an electrochemiluminescence immunoassay (ECLIA) on Modular Analytics E170 (Roche Diagnostics GmbH, Mannheim, Germany). Plasma levels of Sex Hormone Binding Globulin (SHBG) were measured by chemiluminescent immunometric assay (CLIA) on Immulite^® ^2000 (Diagnostic Products Corporation, Los Angeles, CA, USA). The respective detection limits and analytic coefficients of variation (CV) were 0.018 nmol/L and 5.2% for E_2_; 0.100 IU/L and 2.3% for FSH; 0.095 nmol/L and 6.9% for P_4_; and 0.02 nmol/L and 5.0% for SHBG. For the sake of convenience, SHBG will be referred to as a hormone throughout this paper. According to the laboratory, the postmenopausal reference values for FSH and E_2 _were FSH > 26 IU/L and E_2 _< 0.20 nmol/L. Three measurements of P_4 _were below the detection limit and values were defined as half of the detection limit (0.048 nmol/l). Two measurements of SHBG were above the calibration range, and values were defined as the upper limit of the range (180 nmol/L).

The analysis of Modular Analytics E170 of SHBG is not validated for citrate plasma by the manufacturer. The Department of Medical Biochemistry performed a small verification analysis, using serum and citrate plasma from 21 healthy volunteers (data not published). The results indicated a good correlation between measurements in serum and citrate plasma for all hormones measured (0.9899 ≤ r^2 ^≤ 0.9997).

### Statistical methods

We used SPSS^® ^14.0 for Windows for the statistical analyses. Geometric mean plasma levels across different categories of HRT use or BMI were compared using univariate analysis of covariance (ANCOVA) through the general linear model approach. Additionally, we used multiple linear regression to test the association between BMI (continuous variable) and hormone levels. Covariates tested for potential confounding: age, alcohol consumption (units per week), parity and BMI (ANCOVA across HRT categories). In the analysis of the association between BMI and hormone level among HRT users, we also included HRT category as a potential confounder. With the exception of age, only covariates that contributed significantly to the model were included in the final analysis. Time since menopause was excluded as a covariate, due to 25% missing values among postmenopausal women. Sidak corrected post hoc comparisons were used to determine which group means differed. Levene's homogeneity-of-variance test was used to check the equality of group variances. The association between natural log-transformed hormone levels and time since menopause were tested by partial correlation, controlling for BMI. Difference in hormone levels according to time since last HRT dose (0 or 1 day) or fasting (≥10 hours since last meal [10]), and difference in SHBG level between the use of oral and other HRT regimens were analysed with Student's *t*-test for independent samples. We used the McNemar's test for correlated proportions to check for differences in sensitivity and specificity between the two questionnaires [20]. All p-values are two-tailed and the level of statistical significance is 5%.

BMI was categorized as underweight (<18.5 kg/m^2^), overweight (≥25.0 kg/m^2^) and obesity (≥30.0 kg/m^2^) [21]. The two lowest categories (underweight and normal weight, <25.0 kg/m^2^) were merged, due to there being few underweight women.

Menopausal status at blood draw was determined for each woman, based on her answers in the two-page questionnaire as to whether she still had regular menstrual periods, whether the periods were irregular or whether they had stopped. Women were classified as postmenopausal if their periods had stopped and premenopausal if their periods were regular. Women with irregular menses were classified as postmenopausal if they were 53 years or older. This cut-off point was used in a previous NOWAC report [14], based on the definition used in the MWS [15]. The eight-page questionnaire additionally included questions regarding the reason why periods had stopped (natural stop, bilateral oophorectomy, hysterectomy or other reasons) and the age when periods had stopped. When classifying according to plasma levels, we used the postmenopausal reference values, both FSH > 26 IU/L and E_2 _< 0.20 nmol/L, as cut-off. Women with either high FSH or low E_2 _levels were not classified as postmenopausal. The menopausal classification used as a basis for the between group analyses is a combination of these classification procedures. We used the plasma level classification as the gold standard to validate self-reported menopausal status defined by each questionnaire.

HRT use was categorized according to E_2 _content: no HRT, HRT without E_2 _(i.e. estriol, tibolone and other progestogens), E_2 _for vaginal application, E_2 _patches (all dosages), oral 1 mg E_2 _(continuous and sequential preparations), and oral 2 mg E_2 _(continuous, but also sequential preparations with 1 mg E_2 _in 6 out of 28 tablets).

To assess the validity of HRT use, we compared plasma E_2 _levels among HRT users with the 95% CI for plasma E_2 _levels among postmenopausal non-users, and we examined to what extent plasma E_2 _levels among non-users exceeded 0.20 nmol/L.

## Results

**Figure 2:**
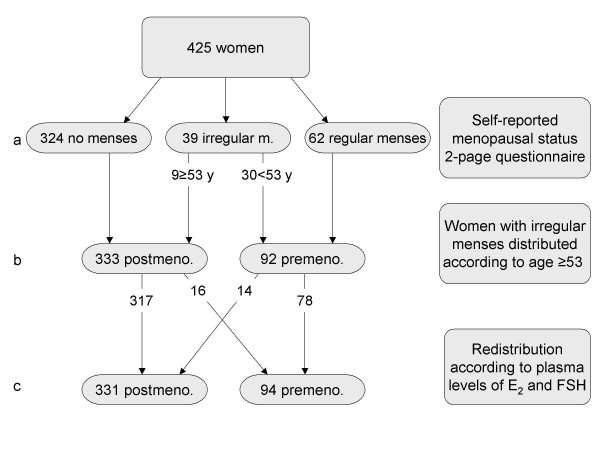
**Flow chart of menopausal status classification in the study sample**. Level c: 16 postmenopausal women not using HRT had premenopausal plasma levels of E_2 _and FSH, including 2 of the 9 women reporting irregular menses, while 14 of the 30 women reporting irregular menses had postmenopausal plasma levels.

Table 1 shows an overview of population characteristics. Based on self-reported age when their periods stopped, 90% of postmenopausal women experiencing natural menopause were postmenopausal by the age of 53. Figure 2 shows the combined classification of menopausal status.

**Table 1:** Characteristics of the study population

	Postmenopausal*			Pre/perimenopausal*			Total		
	N	Mean	95% CI	N	Mean	95% CI	N	Mean	95% CI
Age (years)	331	55.8	(55.4–56.2)	94	50.1	(49.6–50.5)	425	54.5	(54.1–54.9)
Years since menopause	247	8.1	(7.4–8.2)	-	-	-	-	-	-
BMI (kg/m^2^)	331	25.6	(25.1–26.0)	93	25.2	(24.2–26.1)	424	25.5	(25.1–25.9)

* Defined by the combined classification based on both questionnaires and hormone levels (Fig. 2 level c)

### Hormone levels according to HRT use among postmenopausal women

Of 331 postmenopausal women, 66 were current HRT users. Among oral preparations, 84% were combinations of E_2 _and norethisterone actetate (NETA), while 16% were E_2_-only preparations. Most patches were E_2_-only preparations, except one woman using a transdermal combination of E_2 _and NETA. Three of the 66 users showed signs of non-compliance, based on their reported date of last HRT dose. Six women did not report the date.

**Figure 3:**
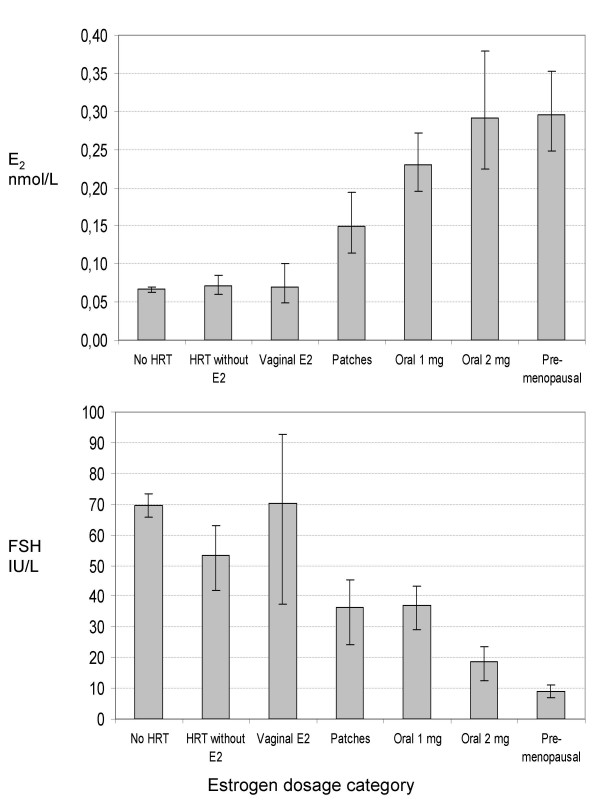
**Plasma levels of estradiol and FSH according to estradiol dosage and rout of administration**. Geometric mean ± 95% CI, premenopausal levels are included for comparison.

Table 2 shows the distribution of women and geometric mean plasma levels of hormones for each HRT user category. The plasma levels of E_2 _increased with increasing E_2 _dose (Table 2, Figure 3). Moreover, use of systemically-administered HRT (patches and tablets) containing E_2 _suppressed the level of FSH (Table 2, Figure 3). There were statistically significant differences in P_4 _and SHBG levels across categories of HRT use (Table 2), although not in a dose-dependent manner in the case of P_4_. The assumption of homogeneity of variance was violated in the analysis of E2 and FSH (Levene's test p < 0.01). The ratio between highest and lowest variance was 7.6 for E2 and 8.7 for FSH.

**Table 2:** Geometric mean plasma levels^3 ^of E_2_, FSH, P_4 _and SHBG according to use of HRT among postmenopausal women

E_2 _dosage category		E_2_^¤ ^nmol/L	FSH^¶ ^IU/L	P_4 _nmol/L	SHBG^‡ ^nmol/L
	n	95% CI	95% CI	95% CI	95% CI
No HRT	265^1^	0.07 (0.06–0.07)	69.6 (65.9–73.5)	0,82 (0.76–0.89)	42.4 (40.2–44.8)
HRT					
HRT without E2	20	0.07 (0.06–0.08)	53.4 (44.0–64.9)	0,65 (0.49–0.87)	34.9 (28.7–42.4)
Vaginal E2	5	0.07 (0.05–0.10)	70.4 (47.8–104)	0,99 (0.56–1.76)	33.4 (22.6–49.4)
Patches	9	0.15 (0.12–0.19)	36.4 (27.3–48.5)	1,05 (0.68–1.61)	47.0 (35.1–62.8)
Oral 1 mg	23^2^	0.23 (0.20–0.27)	36.9 (30.6–44.6)	0,46 (0.35–0.60)	52.1 (43.4–62.5)
Oral 2 mg	9	0.29 (0.22–0.38)	18.7 (14.1–25.0)	0,79 (0.51–1.21)	62.5 (46.8–83.6)
ANCOVA^3^		p < 0.01	p < 0.01	p < 0.01	p < 0.01

^1^FSH: n = 252

^2 ^FSH: n = 21

^3 ^Covariates included in addition to age: ^‡ ^BMI, ^¶ ^BMI and alcohol, ^¤ ^BMI and BMI*age interaction term. Parity did not contribute significantly to the model for any of the analyses, and was omitted.

The post hoc comparisons showed that there was no difference in plasma E_2 _level between vaginal E_2 _application and no E_2 _use (i.e. no HRT use and HRT without E_2_). The main difference was between no/vaginal E_2 _use and systemically administered HRT (p ≤ 0.01). The same tendency was seen for FSH levels, although not as conclusive as for E_2 _levels. The post hoc comparison did not reveal any systematic pattern of differences for P_4 _and SHBG levels across HRT categories. There was, however, a statistically significant difference (p = 0.02) in SHBG level between use of oral HRT (54.5 nmol/L, 95% CI: 45.8–64.9) and the other HRT users (39.8 nmol/L, 95% CI: 32.6–48.6). There was a borderline significant difference in E_2 _level (p = 0.05) between those who had taken their last tablet on the day of blood sampling (n = 17) and those who had taken their last tablet the day before (n = 11).

### BMI and hormone levels

There was no significant difference in BMI between premenopausal and postmenopausal women (Table 1). Among postmenopausal women there was no significant difference in BMI between HRT users (25.1 kg/m^2^, 95% CI: 24.0–26.2) and non-users (25.7 kg/m^2^, 95% CI: 25.2–26.2) (p = 0.30).

Among non-users, there was a significant difference in FSH and SHBG levels across the three categories of BMI, but not in E_2 _or P_4 _levels (Table 3). The regression coefficients (β) showed that one unit increase in BMI was significantly associated with a -3.5% decreased FSH level (95% CI: -4.5% – -2.4%), a -5.4% decreased SHBG level (95% CI: -6.6% – -4.3%), and a 1.6% increased E_2 _level (95% CI: 0.5% – 2.7%), after adjusting for age. Among HRT users (data not shown), there was significant association between BMI and FSH (p < 0.01), but not between BMI and the other hormones analysed (adjusted for age and estrogen dosage category).

**Table 3:** Geometric mean plasma levels* of E_2_, FSH, P_4 _and SHBG according to BMI among postmenopausal women not using HRT

		E_2 _nmol/L	FSH IU/L	P_4 _nmol/L	SHBG nmol/L
	n	95% CI	95% CI	95% CI	95% CI
BMI (kg/m^2^)					
BMI < 25	137	0.07 (0.06–0.07)	76.7 (71.8–81.9)	0.87 (0.78–0.97)	51.8 (48.2–55.7)
25 ≤ BMI < 30	91	0.07 (0.06–0.07)	66.3 (61.2–71.9)	0.78 (0.68–0.89)	35.9 (32.8–39.2)
BMI ≥ 30	37	0.08 (0.07–0.09)	52.0 (45.8–59.1)	0.74 (0.61–0.91)	29.1 (25.3–33.5)
ANCOVA*		p = 0.08	p < 0.01	p = 0.25	p < 0.01

* Adjusted for age. The covariates alcohol and parity did not contribute significantly to the model for any of the analyses, and was omitted.

### Hormone levels among postmenopausal women not taking HRT

There was negative correlation between time since menopause and plasma E_2 _levels (r = -0.16, p = 0.03) and P_4 _(r = -0.29, p < 0.01), and positive correlation between SHBG levels and time since menopause (r = 0.15 and p = 0.04). FSH levels were not significantly correlated with time since menopause.

There was no statistically significant difference in hormone levels between the samples received within 24 hours and those transported over 2, 3 or 4 or more days among either pre-/perimenopausal or postmenopausal women (data not shown). Similarly, there was no statistically significant difference in plasma level between fasting and non-fasting subjects for any of the hormones measured, and lipaemia (n = 13) and haemolysis (n = 30), encountered by visual examination, did not influence the hormone levels (data not shown).

### Premenopausal women

**Figure 4:**
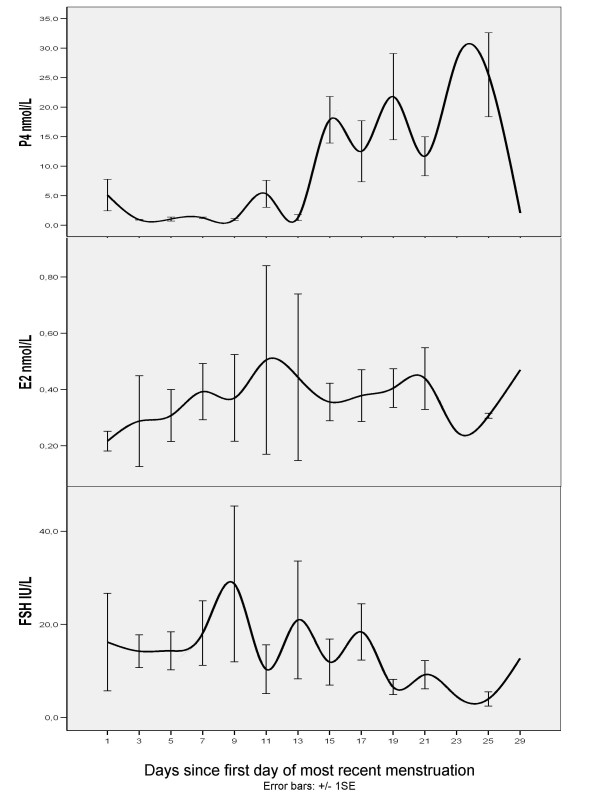
**Plasma levels of progesterone, estradiol and FSH according to days since most recent menstruation among 62 premenopausal women**. Geometric mean ± 1SE, 2 period moving average.

Figure 4 shows plasma levels of E_2_, P_4 _and FSH, according to days since menstruation among premenopausal women who reported having regular periods and who filled in the date of the first day of their most recent menstruation (n = 62). Although there were few women in each two-day period (n = 1–8), the pattern of hormonal variation throughout the menstrual cycle was recognizable both for the gonadal hormones and FSH. Progesterone levels > 20 nmol/L were only found among women in their luteal phase (≥15. day). There was no recognizable cyclic hormone pattern among the 20 perimenopausal women who had reported their menstruation date. One woman used an oral contraceptive, a progestagen-only pill.

### Validation of self-reported questionnaire information on menopausal status and hormone use

Sensitivity and specificity for the variable "menopausal status" defined by the two questionnaires used is shown in Table 4. The 66 HRT users were excluded from this analysis. Sensitivity was higher in the two-page questionnaire accompanying the blood sample (p < 0.05). The eight-page questionnaire scored higher on specificity (p < 0.01).

**Table 4:** Validation of the menstrual status definition according to questionnaire information

	Classification according to plasma E_2 _and FSH levels		
	Post-	Pre-	Total
Classification according to two-page questionnaire			
Post-	240	27	267
Pre-	20	72	92
Total	260	99	359
Sensitivity: 240/260 = 92% (95% CI 89–96%)			
Specificity: 72/99 = 73% (95% CI 64–82%)			

Classification according to eight-page questionnaire			
Post-	229	11	240
Pre-	29	86	115
Unknown	2	2	4
Total	260	99	359
Sensitivity: 229/260 = 88% (95% CI 84–92%)			
Specificity: 86/99 = 87% (95% CI 80–94%)			

Out of 41 self-reported users of systemically-administered E_2 _preparations, 39 women (88%) had E_2 _levels above the confidence interval of non-users. Thus, self-reported HRT use was confirmed by the hormone levels. Among the 265 self-reported non-users, seven women had E_2 _levels ≥ 0.20 nmol/L. According to the preceding eight-page questionnaire, six out of the seven women reported being pre- or perimenopausal or uncertain regarding menopausal status six months before the blood draw. At the same time, they also reported being non-users, they did not state their age at menopause, and all seven were younger than 53 years old. Hence, they were probably perimenopausal at the time of blood draw, and not misclassified HRT users. On the basis of this, we conclude that the specificity of the questionnaire variable "current HRT use" is 100%.

Among 78 women with uncertain menopausal status due to hysterectomy, use of HRT, etc., who according to the eight-page questionnaire were defined as postmenopausal, based on age ≥ 53 years, 4 were misclassified according to plasma hormone levels.

## Discussion

The associations found between current HRT use and plasma levels of E_2 _confirm previous reports [10-12]. Transdermal E_2 _50 μg/day, which is the Defined Daily Dose (DDD) [22] for E_2 _patches, should give approximately the same plasma E_2 _levels as oral 1 mg E_2_. We merged use of patches into one category because there were only 9 users; 5 did not report the dose and the remaining 4 used 50 μg/day or less. Assuming that women in 2005 used the lowest possible dose, it is likely that the average dose among these women was lower than 50 μg/day, and that the plasma levels of E_2 _should be lower for patches than for oral 1 mg E_2_. Users of HRT for vaginal application were not expected to differ much from non-users regarding plasma level of E_2_. The vaginal tablet is applied twice a week (maintenance dosage) and reports from clinical trials show that blood E_2 _levels remain within the normal range of postmenopausal women, even with long-term treatment [23,24]. The confidence intervals of the three systemically-administered HRT groups are partly overlapping, reflecting insufficient power to conclude that the plasma levels are different.

The increase in FSH levels as a woman approaches menopause is a result of reduced ovarian inhibin synthesis and increased activin synthesis [25], and it has been assumed that FSH is not influenced by exogenous estrogen supplements during menopause [26]. Pharmacokinetic studies of HRT rarely measure FSH levels, nor did NHS [10] in their population-based study on HRT users. However, there are some reports which show a decrease in FSH levels during the long-term use of both oral [27,28] and transdermal E_2 _[29]. Our results suggest that exogenous E_2 _has some effect on FSH levels.

It was not expected that P_4 _levels would be influenced by HRT use. Although we found differences in P_4 _levels across categories of HRT use, there was no association with E_2 _dosage. For the purpose of validation, it would have been rational to measure plasma levels of NETA rather than P_4 _among HRT users, however this was not feasible with our available methods at the time of analysis.

During daily use of oral HRT, the half life of E_2 _at a steady state is 15–25 hours, due to sequestration in adipose tissue and enterohepatic recycling [30]. Several reports also show that the use of E_2 _preparations increases the SHBG plasma concentration, which would increase the blood depot of E_2_. However, this applies to the use of CEE to a greater extent than 17-β-estradiol preparations [31,32]. In addition, oral rather than topical preparations seem to increase SHBG levels, due to the liver first pass effect [11,12,31,32]. This is in accordance with our results, showing that differences in SHBG levels across HRT categories are mainly due to significantly increased levels among the users of oral preparations.

The results on the correlation between time since menopause and levels of E_2_, FSH and SHBG are in accordance with previous reports [33,34]. Plasma FSH levels are already high at menopause, while the E_2 _levels are still dropping.

The lack of association between BMI and HRT use supports conclusions from previous reports [35-37]. The association found between BMI and hormone levels is also in accordance with established knowledge. The absence of a stronger relationship than the one found between E_2 _and BMI may be due to the assay used. There are reports showing that direct immunoassays are hampered by insufficient sensitivity and specificity when measuring low concentrations of steroids, e.g. E_2 _among postmenopausal women [38]. If the E_2 _levels are artificially high in the low range, this would weaken the association. It could also be the reason why the ANCOVA did not show any significant increase across BMI categories, or alternatively this could be due to lack of power. We may also suggest a possible effect of BMI in increasing the bioavailability of estradiol through lower levels of SHBG, which is in accordance with previous findings [6,13]. We did not measure estrone (E_1_), which may be a better biomarker of estrogen synthesis in adipose tissue among postmenopausal women, although levels of endogenous estradiol and estrone are highly correlated [6,39].

Using plasma levels of sex hormones to define a woman as premenopausal would be difficult, due to the variation in hormone levels throughout the menstrual cycle. In addition, the menstrual cycle changes as the woman approaches menopause [33,34,40]. The follicular phase shortens; FSH levels begin to increase, and E_2 _levels subsequently decrease. Although we are not trying to validate the self-reported day of menstrual cycle, the fact that the cyclic pattern of plasma sex hormones is visible at all in this rather small group of older premenopausal women provides some assurance in this matter.

We have used plasma levels of E_2 _and FSH as the gold standard in our validation of self-reported questionnaire information on HRT use and menopausal status. Whether the gold standard chosen is a proper gold standard is always debatable. However, hormone levels were considered to be the best available instrument. To draw the line between pre- and postmenopause, we have used plasma levels of FSH > 26 IU/L and E_2 _< 0.20 nmol/L as the cut-off point. Several clinical trials conducted among postmenopausal subjects use FSH levels > 30–50 IU/L and/or E_2 _levels < 0.07–0.15 nmol/L as inclusion criteria [11,12,30]. These rather strict criteria are imperative in clinical trials, to ensure that all the participants are postmenopausal. However, this generates a rather large group of false negatives, which is inappropriate to a validation. Furthermore, since the premenopausal E_2 _and FSH plasma levels for 14 out of the 16 women reclassified as premenopausal may be explained from information from the eight-page questionnaire (hysterectomy, use of hormone containing intrauterine device, etc.), our cut-off point seems to be well-chosen.

Since the plasma E_2 _levels among women using vaginal E_2_-preparations were no different from those of non-users, our validation of the variable "current HRT use" had to be based on the women using systemically-administered E_2_-containing preparations. However, we see no reason why the route of administration should influence the women's ability to answer the question, and have extended the result to include all HRT use. The agreement found between self-reported HRT use and plasma E_2 _levels supports previous findings from validation studies comparing self-reported HRT use with prescription data [41,42].

It makes sense to find a lower sensitivity for the eight-page questionnaire, because some women could have become postmenopausal during the six-month lapse until the blood draw. On the other hand, the specificity for the eight-page questionnaire may be artificially high, since some of the premenopausal women will be reported as postmenopausal on the two-page questionnaire six months later, although they are probably in a perimenopausal state. Due to the low misclassification rate, it seems reasonable to continue using age ≥53 as the cut-off point when defining menopausal status in cases of uncertainty.

### Strengths and limitations

The NOWAC study has the advantage of being population-based and prospective. The opportunity of random sampling from the complete Central Population Register, together with high response rates, provides a representative sample of the Norwegian female population aged 48–62 years [43]. The two-page questionnaire accompanying the blood sample provides detailed and updated information on central variables such as menstruation status, weight and HRT use, which are complementary to the preceding eight-page questionnaire. Results show that it is especially valuable to be able to differentiate between different types of HRT. The effect of HRT exposure will vary between populations using different HRT regimens, particularly with respect to estrogenic carcinogenicity.

Compliance with HRT is an important determinant for the accurate measurement of HRT use. With 86–95% of the women showing compliance with HRT use, we do not consider lack of compliance to be a significant source of bias in our study.

Probably due to small sample sizes, the assumption of equal variances was violated in the ANCOVA analysis of differences between HRT categories. This causes some concern and the results must be interpreted accordingly. Even so, the very low p-values suggest that the results for E2 and FSH are valid.

Reports on the validity of self-reported body-size generally show that particularly obese people tend to underestimate their weight/waist circumference [44,45]. In the present study, there is an increase in weight (mean: +0.6 kg) between the eight-page questionnaire and the two-page questionnaire six months later and the increase is higher among the women who had their weight measured at their general physician's office (n = 49). This should not influence our analyses to the degree that the associations found are artefacts. However, if the BMI is under-estimated, the change in hormone levels per increased unit of BMI could be over-estimated.

Choosing plasma or serum is a trade-off between rational collection logistics and the broadest assortment of feasible analyses. Not all general physicians' offices have equipment for blood centrifugation, and at the beginning of the NOWAC blood specimen collection in 2002 the range of future analyses had not yet been determined. With the aim of collecting blood from as many participants as possible, it was decided to build a plasma biobank. Citrate was chosen as the anticoagulant, due to the collaboration of NOWAC with EPIC and their collection of citrate plasma. Citrate plasma is not the optimum matrix for immunometry analyses. Although the verification analysis performed by the laboratory did not show alarming inconsistencies, the results must be interpreted accordingly, especially in view of the general limitations of direct immunoassays mentioned above.

A transport delay of over two days could potentially interfere with our measurements. Several studies have concluded that sex hormones FSH and SHBG are fairly stable with regard to transport conditions, temperature variations and delayed processing [46-50]. These reports are not based on an analysis of citrate plasma. However, in our sample there were no statistically significant differences due to transport delay, and we did not exclude any blood samples on these grounds. The analytical methods used are fairly robust with regard to interference by haemoglobin and triglycerides, and since we found no differences due to lipaemia or haemolysis these samples were not excluded from the analysis. Due to the study design, blood samples were not drawn at the same time of day, nor were the women requested to be fasting. However, we found no differences in hormone levels due to fasting and have not adjusted our results according to time since last meal.

Because of the cross-sectional nature of this study, we cannot draw any conclusions regarding causal association based on our results. However, imminent gene-expression analyses and future follow-up of the women in our population sample will contribute to our knowledge on causal relationships.

## Conclusion

Users of systemically-administered E_2_-containing HRT preparations have plasma E_2 _and FSH levels comparable to those of premenopausal women, while users of vaginal E_2 _preparations remain within postmenopausal levels. There is no difference in BMI between HRT users and non-users, but increased BMI is associated with increased E_2 _and decreased FSH and SHBG levels among non-users. The NOWAC questionnaires provide valid information on current hormone use and menopausal status among Norwegian women who are 48–62 years old.

## Competing interests

The author(s) declare that they have no competing interests.

## Authors' contributions

MW, VD and EL participated in the design of the study. MW performed the statistical analysis and drafted the manuscript. VD, KB, KSD, CR and EL helped to draft the manuscript. YF was in charge of the immunoassays. EL is the principal investigator of the NOWAC study. All authors read and approved the final manuscript.

## Pre-publication history

The pre-publication history for this paper can be accessed here:


